# Supplementary material for: Core clock genes adjust growth cessation time to day-night switches in poplar
Source: Nat Commun. 2024 Feb 27;15:1784. doi: 10.1038/s41467-024-46081-6 (PMC10899572; doi:10.1038/s41467-024-46081-6)
Supplement: Supplementary file 1 — Supplementary Information [file 41467_2024_46081_MOESM1_ESM.pdf]

Supplementary Information for

Core Clock Genes Adjust Growth Cessation Time to Day-Night Switches in Poplar.

Daniel Alique, Arturo Redondo López, Nahuel González Schain, Isabel Allona, Krzysztof Wabnik and Mariano Perales

Contents

Supplementary Figure 1. Simulated *FT* transcription patterns in *Arabidopsis* mutants.

Supplementary Figure 2. Daily transcription patterns of studied photoperiodic regulators in LD and SD.

Supplementary Figure 3. Daily transcription patterns of studied photoperiodic regulators orthologs in *Arabidopsis* under LD and SD.

Supplementary Figure 4. CRISPR-Cas9 mutations targeting TOC1 and GI.

Supplementary Figure 5. Daily transcription patterns of studied photoperiodic regulators under LD, comparing WT to CRISPR-Cas9 lines targeting TOC1, GI, and LHY2.

Supplementary Figure 6. Conserved cis/trans-regulatory elements for the suggested TOC1 repression of *FT2* transcription.

Supplementary Figure 7. Simulated *FT2* expression considering CDF2 (instead of LHY2) as second repressor in the computational model.

Supplementary Figure 8. Predicted *FT2* expression window under different daylengths.

Supplementary Figure 9. Increased LHY2 expression after night extension predicts *FT2* downregulation.

Supplementary Figure 10. Sensitivity analysis of predicted *FT2* expression deviation after altering the input gene Gaussian pulses.

Supplementary Table 1. Primers used in this work.

Supplementary Table 2. Parameter description and value for the *FT2* transcription models.

Supplementary Note 1. Description of the *FT2* transcription computational model.

Supplementary References

SUPPLEMENTARY FIGURES

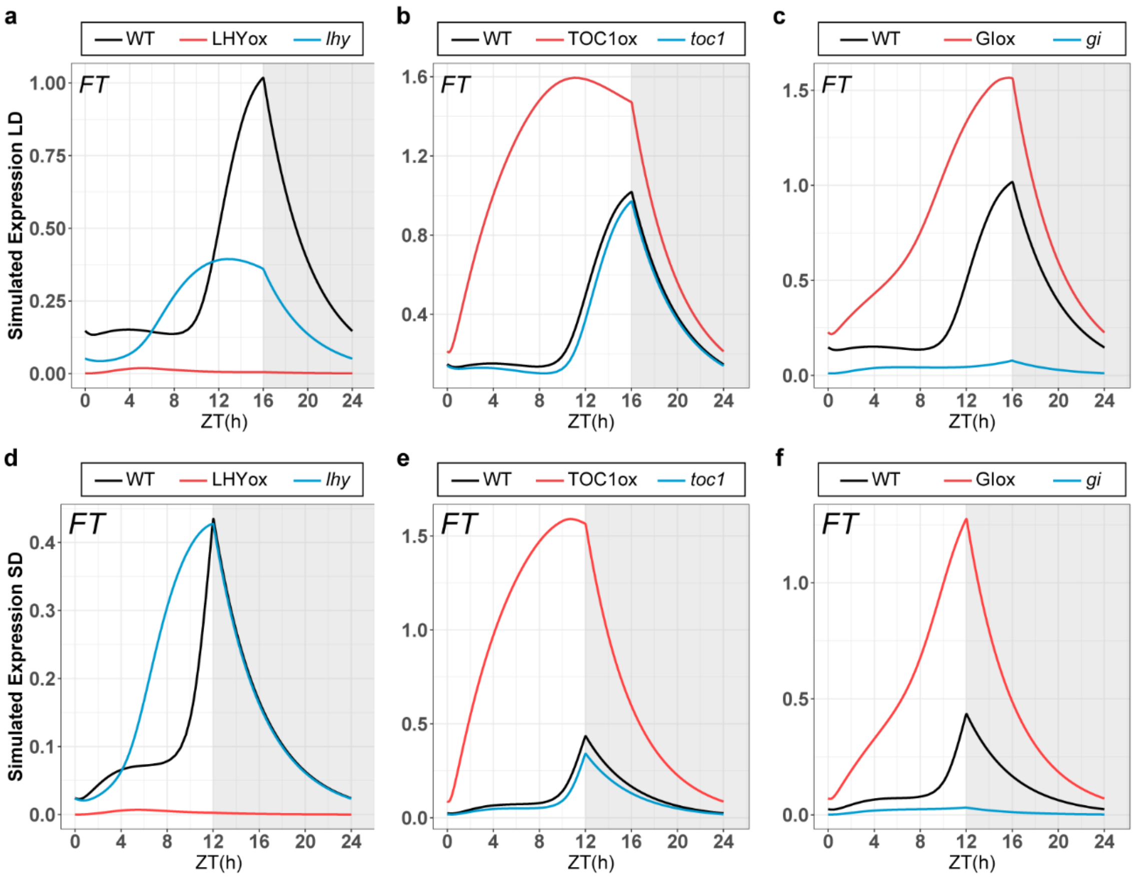

Supplementary Figure 1. Simulated *FT* transcription patterns in *Arabidopsis* mutants.

(**a**-**f**) Simulated *FT* levels for LHY (a,d), TOC1 (b,e), and GI (c,f) gain (red) and loss-of-function (blue) lines in *Arabidopsis*, compared to WT (black). These simulations are based on a previously published model[1] and are shown for LD 16h light: 8h dark (a-c) and SD 12h light: 12h dark (d-f). To simulate overexpression of TOC1 (b,e) and GI (c,f) (not reported before), we introduced an additional basal expression parameter (b=5) into the respective equation. ZT, Zeitgeber Time in hours.

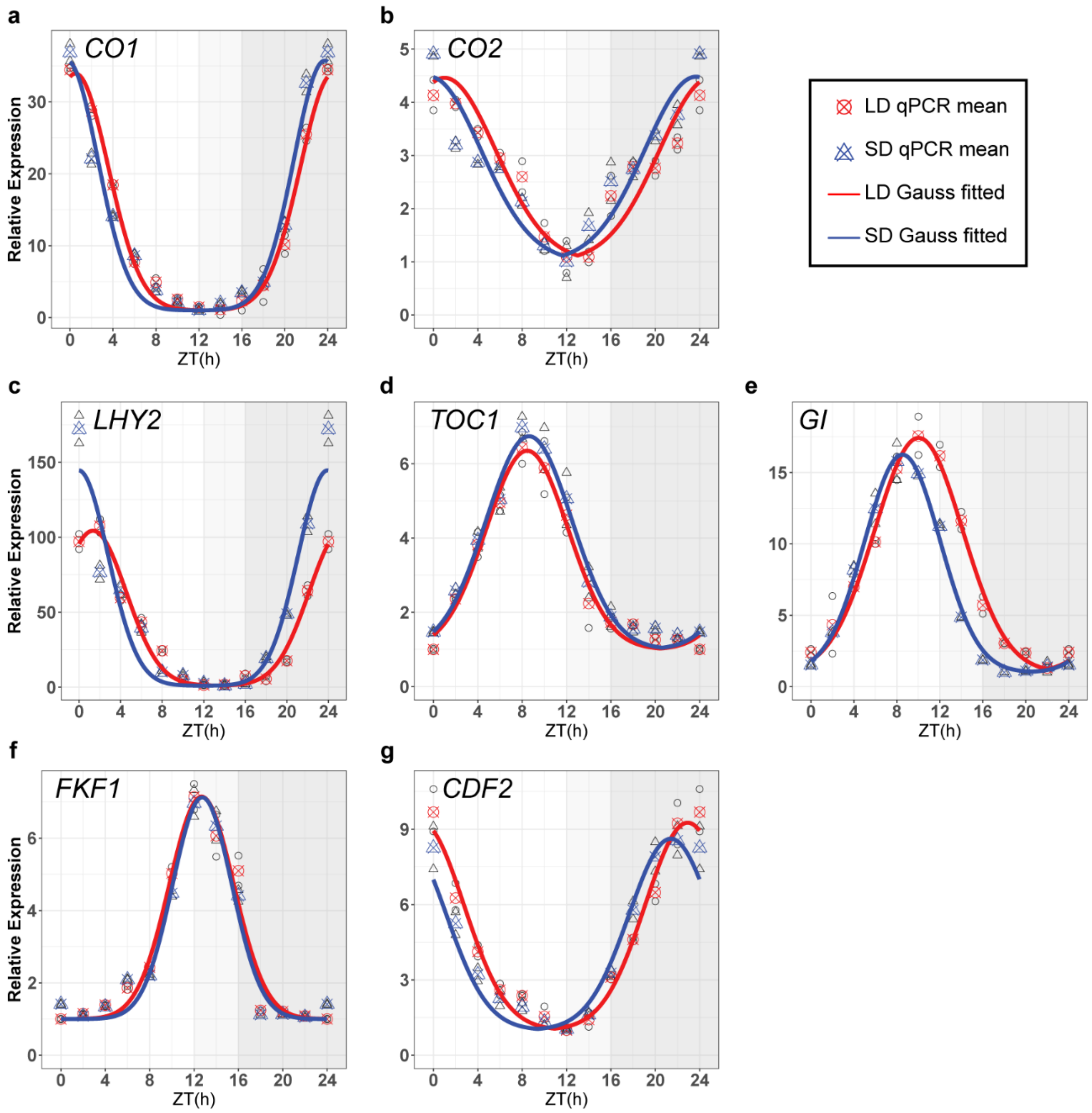

Supplementary Figure 2. Daily transcription patterns of studied photoperiodic regulators in LD and SD.

(**a-g**) qPCR results are shown for LD (circles) and SD (triangles), with the means of the two biological replicates used for fitting highlighted - crossed and colored in red (LD) and blue (SD). Solid lines represent fit to a Gaussian function. Grey boxes indicate nightlength (light grey only in SD). ZT, Zeitgeber Time in hours. Related to Figures 1d and 1e.

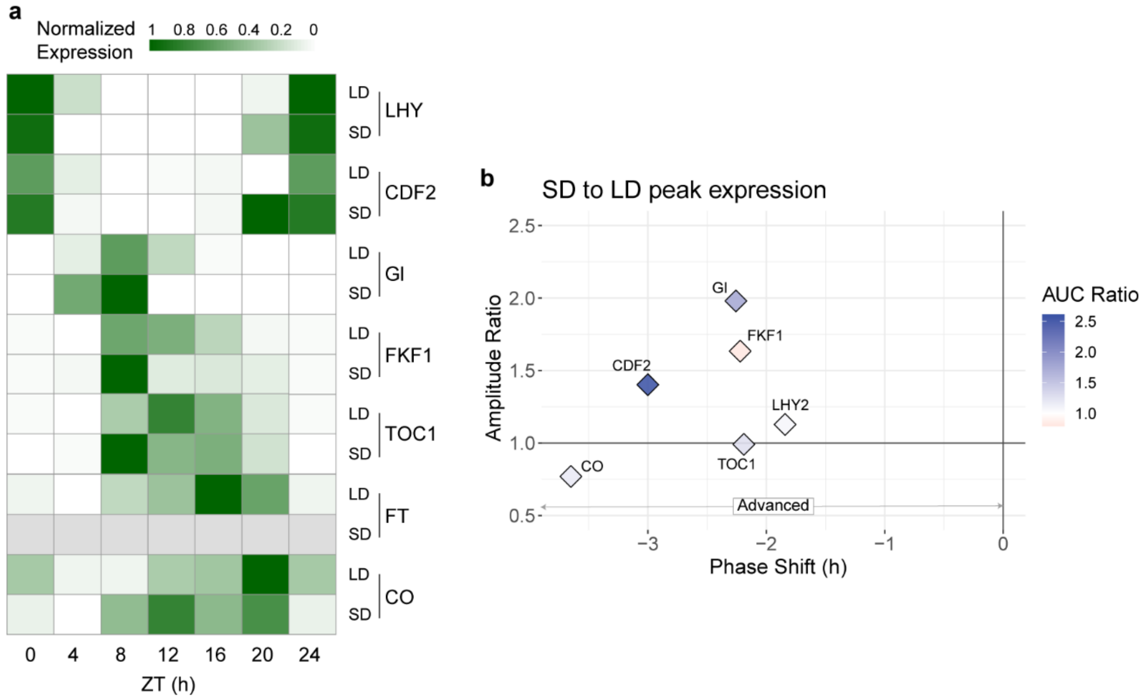

Supplementary Figure 3. Daily transcription patterns of studied photoperiodic regulators orthologs in *Arabidopsis* under LD and SD.

(**a**) Heat map of daily expression in LD and SD. From Microarray results[2] first day of expression was considered and the patterns were normalized 0-1 per gene. *FT* in SD is shown in grey due to non-detection. ZT, Zeitgeber Time in hours. (**b**) SD to LD deviation of the gene expression patterns shown in (a) for phase (time of maximal expression) shift, amplitude ratio, and daily total transcript ratio (AUC - Area Under the Curve) after Gaussian fit.

a

TOC1

7610 bp

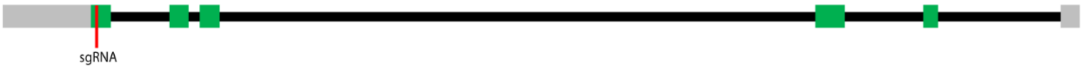

DNA sequences

```
sgRNA              -------------------------------GTGGGCACATGAAGGA-GAG---------------------------------------------------------------
TOC1 P.tremula     ATGGAGGGAGAGGTAGATGAGCAAAATTTGAGTGGGCACATGAAGGA-GAGTGGTAATGGTAAAAGTGTTGGAGGAGCAGGAGCAGGAGCAGGAGATGGTTTTGTTGATAGAAG...
TOC1 P.alba        ATGGAGGGAGAGGTAGATGAGCAAAATTTGAGTGGGCACATGAAGGA-GAGTGGTAATGGTAAAAGTGTAGG-----------AGGATCAGGAGATGGTTTTGTTGATAGAAG...

toc1-2 P.tremula   ATG---------------------------------------------GAGTGGTAATGGTAAAAGTGTTGGAGGAGCAGGAGCAGGAGCAGGAGATGGTTTTGTTGATAGAAG...
toc1-2 P.alba      ATGGAGGGAGAGGTAGATGAGCAAAATTTGAGTGGGCACATGAAGGAAGAGTGGTAATGGTAAAAGTGTAGG-----------AGGATCAGGAGATGGTTTTGTTGATAGAAG...
```

Protein sequences

```
TOC1 P.tremula     MEGEVDEQNLSGHMKESGNGKSVGGAGAGAGDGFVDRSKVRILLCDNDATSSQEVFTLLLKCSYQVTSVRSARQVIDALNAEGPEIDIILSEVDIPMTKGMKMLKYITRDKDLR...
TOC1 P.alba        MEGEVDEQNLSGHMKESGNGKSVGGSGDGFVDRSKVRILLCDNDAKSSQEVFTLLLKCSYQVTSVRSARQVIDALNAEGPEIDIILSEVDIPMTKGMKMLKYITRDKDLQRIPV...

toc1-2 P.tremula   MEW*
toc1-2 P.alba      MEGEVDEQNLSGHMKEEW*
```

b

GI

11495 bp

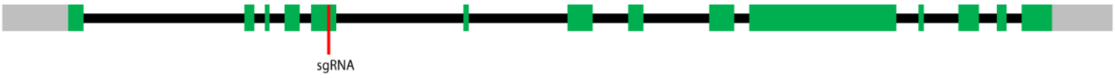

DNA sequences

```
sgRNA                 -----------------------------------------------GAAATCCTCCAGCATGCCTTTGG----------------------------------------------
GI P.tremula       ...AGAAGCAGTAGTGAGAGCCACGCTACAAGTAGTGAATCAGCAGAAGGGAAATCCTCCAGCATGCCTTTGGTACAACAAGAGAGGAAACCTTTCAGGCCTCTGTCTCCCTGGATCAC...
GI P.alba          ...AGAAGCAGTAGTGACAGCCAGGCTACAAGTAGTGAATCAGCAGAAGGGAAATCCTCCAGCATGCCTTTGGTACAACAAGAGAGGAAACCTTTCAGGCCTCTGTCTCCCTGGATCAC...

gi-5 P.tremula     ...AGAAGCAGTAGTGAGAGCCACGCTACAAGTAGTGAATCAGCAGAAGGGAAATCCTCCAGCATG----TGGTACAACAAGAGAGGAAACCTTTCAGGCCTCTGTCTCCCTGGATCAC...
gi-5 P.alba        ...AGAAGCAGTAGTGAGAGCCACGCTACAAGTAGTGAATCAGCAGAAGGGAAATCC-------------TGGTACAACAAGAGAGGAAACCTTTCAGGCCTCTGTCTCCCTGGATCAC...
```

Protein sequences

```
GI P.tremula       ...GEILRILTHYNRPIYKREQQNNETDRSSSESHATSSESAEGKSSSMPLVQQERKPFRPLSPWITDILLAAPLGIRSDYFRWCSGVMGKYAAGELKPPTTTSSRGSGKHPQLIPSTP...
GI P.alba          ...GEILRILTHYNRPIYKREQQNNETDRSSSDSQATSSESAEGKSSSMPLVQQERKPFRPLSPWITDILLAAPLGIRSDYFRWCSGVMGKYAAGELKPPTTTSSRGSGKHPQLIPSTP...

gi-5 P.tremula     ...GEILRILTHYNRPIYKREQQNNETDRSSSESHATSSESAEGKSSSMRYNKRGNLSGLCLPGSPIYCLLHLWVLEVTTFVGAVVLWVNMLRESSNHQQPLLHVGLENILSSSHQHQGGL*
gi-5 P.alba        ...GEILRILTHYNRPIYKREQQNNETDRSSSDSQATSSESAEGKSWYNKRGNLSGLCLPGSPIYCLLHLWVLEVTTFVGAVVLWVNMLRESSNHQQPLLHVGLENILSSSHQLQGGL*
```

Supplementary Figure 4. CRISPR-Cas9 mutations targeting TOC1 and GI.

(**a**,**b**) Schematic representation of TOC1 (a) and GI (b) genes, with the localization of the sgRNA (red) used for CRISPR-Cas9 editing. 5'UTR and 3'UTR are shown in grey, exons in green and introns in black. DNA genomic sequences flanking sgRNA and predicted protein translations obtained for WT and *toc1* (a) or *gi* (b) knockouts in both *alba* and *tremula* haplotypes are presented. sgRNA nucleotides matching the genome are displayed in red. Insertions or deletions are shown in blue. Asterisks indicate the premature stop codons formed in the truncated proteins.

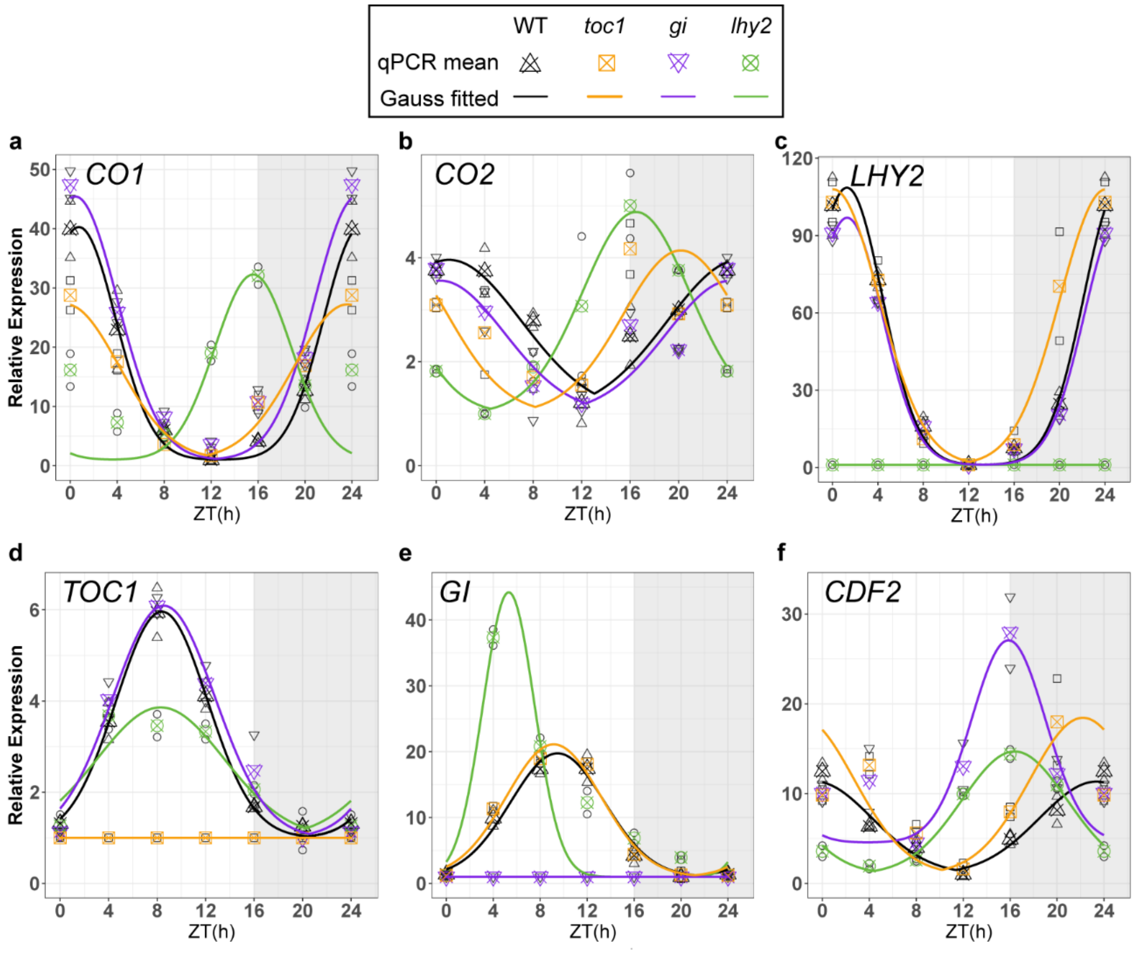

Supplementary Figure 5. Daily transcription patterns of studied photoperiodic regulators under LD, comparing WT to CRISPR-Cas9 lines targeting TOC1, GI, and LHY2.

(**a-f**) Spots represent qPCR results in WT (upward triangles) and knockouts for TOC1 (squares), GI (downward triangles), and LHY2 (circles) under LD condition. Means of the two biological replicates used for fitting are highlighted - crossed and colored in black (WT), yellow (*toc1*), purple (*gi*), and green (*lhy2*). Solid lines indicate fit to a Gaussian function. Grey boxes denote nightlength. ZT, Zeitgeber Time in hours. Related to Figures 2c and 2d.

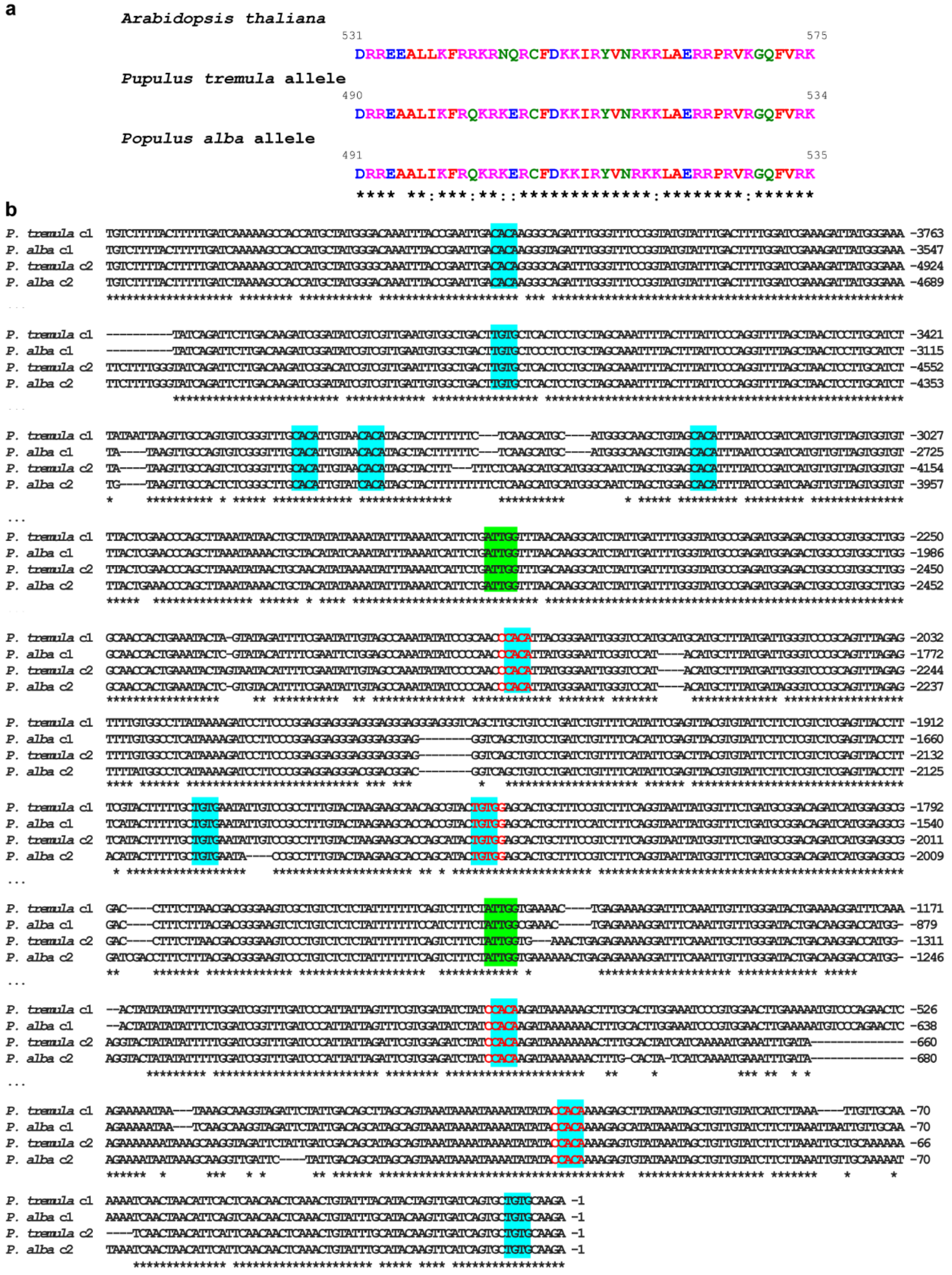

Supplementary Figure 6. Conserved cis/trans-regulatory elements for the suggested TOC1 repression of *FT2* transcription.

(**a**) Sequence alignment of CCT domains in TOC1 homologs. Symbol code: "*" conserved amino acid and ":" conserved amino acid group with high similar properties. (**b**) Conserved cis-elements identified in poplar *FT2* promoters 6kb upstream of the transcriptional start site. T1ME (TGTG) TOC1 binding sites are highlighted in blue[3], CCAAT boxes for NF-YA binding are shown in green[4], and central pentamer TGTGG of the CORE element, identified as CO binding sites *in vitro*, are colored in red[5]. Two *FT2* promoters are shown (c1/2) for haplotype derived from a tandem duplication[6]. (a,b) ClustalW was used for multiple sequence alignments.

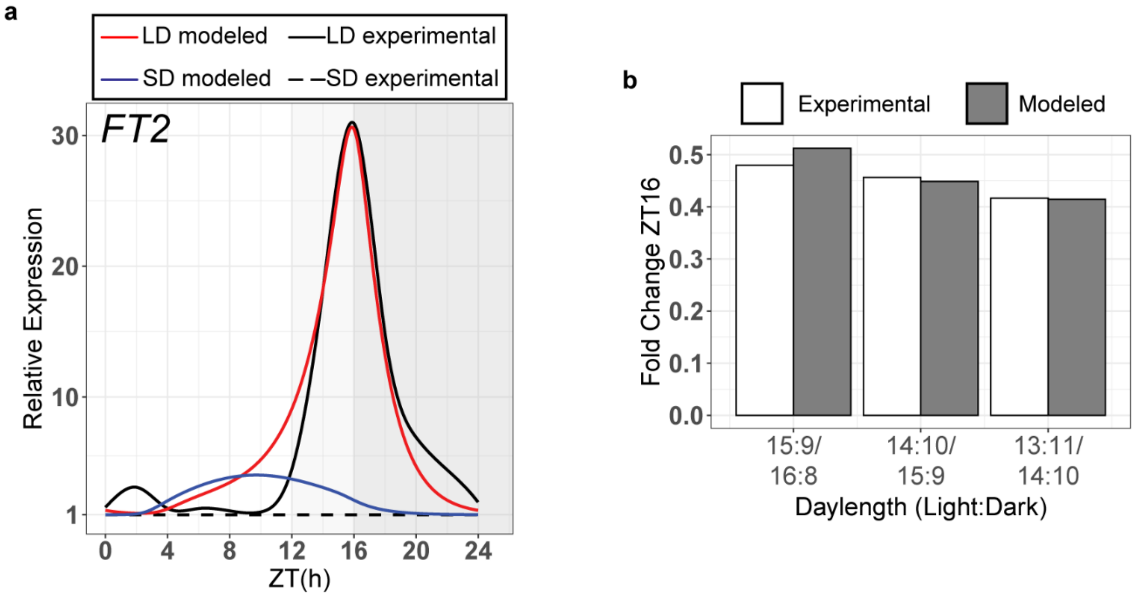

Supplementary Figure 7. Simulated *FT2* expression considering CDF2 (instead of LHY2) as second repressor in the computational model.

(**a**) *FT2* transcription fit (LD-red, SD-blue) to experimental data (spline fitted; LD-black/solid, SD-black/dashed). (**b**) Predicted *FT2* expression fold change at ZT16 for indicated photoperiods (grey bars) compared to experimentally observed (calculated from mean values shown in Figure 1c; white bars). ZT, Zeitgeber Time in hours.

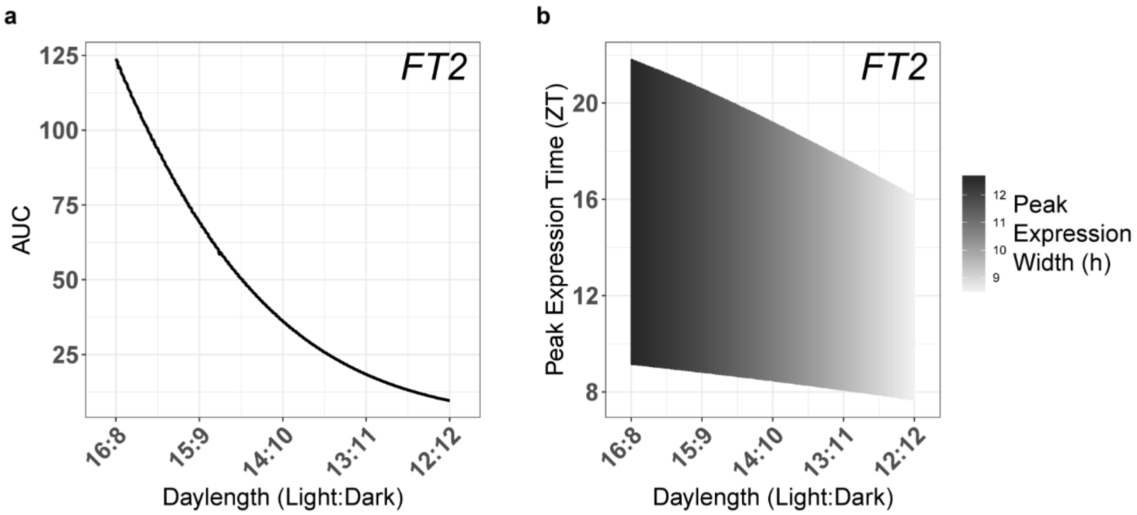

Supplementary Figure 8. Predicted *FT2* expression window under different daylengths.

(**a**) Accumulated transcript for *FT2* expression peak measured as Area Under the Curve (AUC). (**b**) *FT2* peak start and end time points, and width. ZT, Zeitgeber Time in hours. Related to Figure 3e and Supplementary Movie 1.

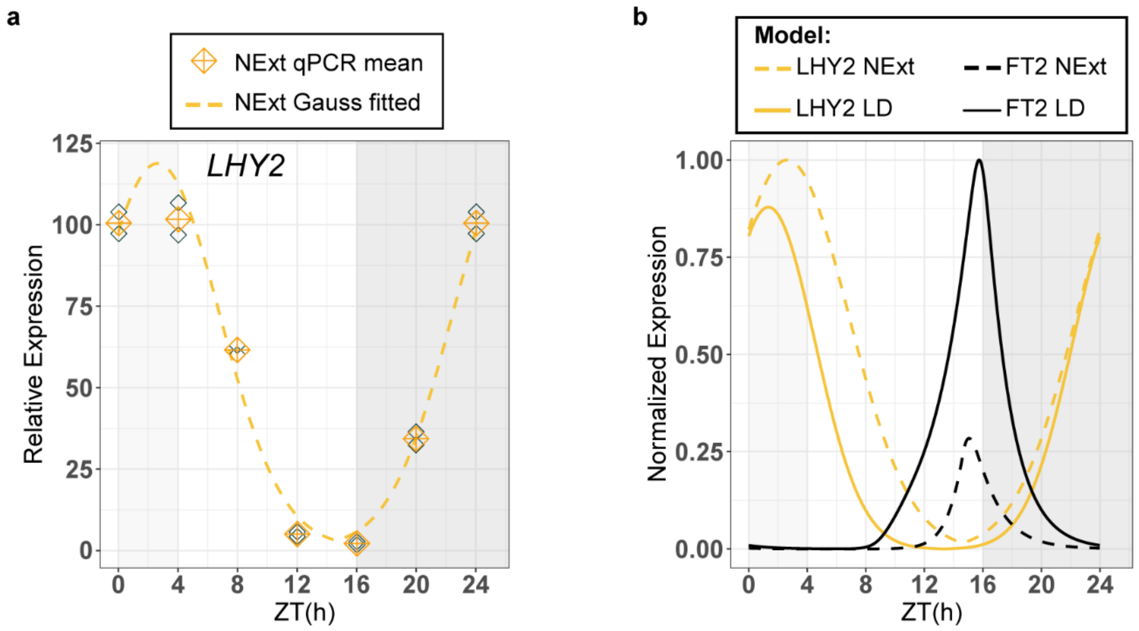

Supplementary Figure 9. Increased LHY2 expression after night extension predicts *FT2* downregulation.

(**a**) Spots represent qPCR results following a 4-hour night extension (Next). Means of the two biological replicates used for fitting are indicated. Solid lines fit a Gaussian function. (**b**) Modeled *LHY2* and *FT2* expression patterns used as input and predicted, respectively, comparing LD and NExt. Gene expression is normalized between 0 and 1. (a,b) Grey boxes depict night length, with light grey only during Next. ZT, Zeitgeber Time in hours.

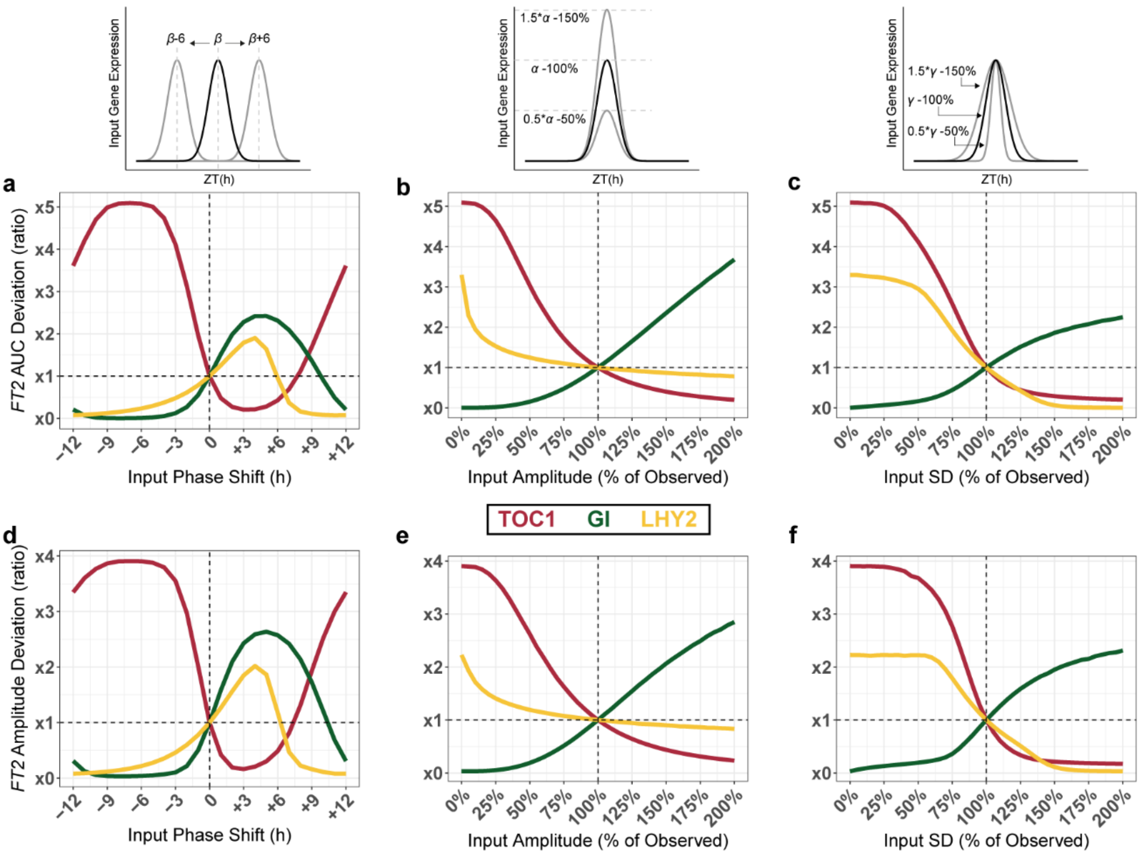

Supplementary Figure 10. Sensitivity analysis of predicted *FT2* expression deviation after altering the input gene Gaussian pulses.

(**a-f**) *FT2* deviations in long-day from best-performing simulation shown in Figure 3b for daily total transcript (AUC – Area Under the Curve; A-C) and amplitude (d-f), following expression pattern modifications in each of the input genes: TOC1 (red), GI (green), and LHY2 (yellow). Top panels illustrate the perturbations applied to the Gaussian function parameters: "*β*" - phase (a,d), "*α"* – amplitude (b,e), and "*γ*" - standard deviation (SD; c,f). Refer to equation 1 in Supplementary Note 1 for details. (a-f) Horizontal dashed line indicates no deviation in FT2 expression pattern (x1), while vertical dashed line displays reference (no input change).

SUPPLEMENTARY TABLES

Supplementary Table 1. Primers used in this work.

**qPCR**		
**Gene**	**FW (5'→3')**	**RV (5'→3')**
**CDF2**	AATGGGTATCATAGACCTGAAC	TCCCTTCTCTGATGAATTTGAA
**CO1**	CAGTGTTCCTCCAAAGAGTTGTGGGG	GATTGTCCCTCTCGGAGCACTTTGGTG
**CO2**	TGGAGTGGTGCCAGAATCAACAGTG	GGTGGCATCTGGATAGCAGTGCTGG
**FKF1**	CGCACACAGAGGGAGAGTGAGG	CTCTTTCCAATTCTTCCTCTTCCTC
**FT2**	CTACCGGGGCGAACTTTGGGCAAGAGGT	TCATGGTCTCCTTCCACCGGAGCCAC
**GI**	AAACATACTCACCTGGACACTGG	AACTTGACGATGCGCTGATTGAC
**LHY2**	GAGGTTTCCAATCCAGGCAA	GGCAGGCAAACGAGGTATG
**TOC1**	GAGCGGCAGACTATCTTGTAAAG	AGATGTTCTTCTCCGCCAATC
**UBQ7**	GGAACGGGTTGAGGAGAAAGAAG	GCAAGAACAAGATGAAGCACAGAGC

**Genotyping**			
**Gene**	**Haplotype**	**Sense**	**Sequence (5'→3')**
**GI**	*tremula*	FW	CCTCCACCTCAAGATGCAAAGAAAGGC
**GI**	*alba*	RV	CACCTCAAGATTTGGGGTGGGGC
**GI**	both		CAGCAGCATCAAGTGCATCTGGAGC
**TOC1**	*tremula*	FW	CTTCATCACCGTCGTCACTTAGGA
**TOC1**	*tremula*	RV	CTGCTCCTGCTCCTGCTC
**TOC1**	*alba*	FW	GCTGTGTTTTCTTCATCACTTAGG
**TOC1**	*alba*	RV	CAACAAAACCATCTCCTGATCCTC

Supplementary Table 2. Parameter description and value for the *FT2* transcription models.

Parameter	Description	Value	
		**LHY2 repressor** (related to Fig. 3)	**CDF2 repressor** (related to Supplementary Fig. 7)
b	*FT2* basal transcription	5.2	
a	GI activation rate	77.8	
ka_{GI}	GI association affinity	0.6	
n_{GI}	GI association cooperativity	3	
r	TOC1 repression rate	160.4	
ka_{TOC1}	TOC1 binding affinity	1	
n_{TOC1}	TOC1 binding cooperativity	3	
$K_{Repressor}$	LHY2/CDF2 repression strength	4.2	3.1
$n_{Repressor}$	LHY2/CDF2 binding cooperativity	3	3
d	*FT2* degradation rate	0.6	

SUPPLEMENTARY NOTES

Supplementary Note 1. Description of the *FT2* transcription computational model.

Data-driven models for *FT2* transcription were developed using MATLAB_R2022a (Mathworks, Cambridge, UK) software following the steps indicated below. Full code of final model with LHY2 as second repressor is provided in Supplementary Data 2. README instructions allow to run the model under different daylengths and in gain- and loss-of-function for the circadian clock genes as shown in Figure 3.

Input genes

qPCR-derived transcriptional patterns of *LHY2*, *GI*, *TOC1*, and *CDF2* in long-day (LD; 16h light: 8h dark) and short-day (SD; 12h light: 12h dark) conditions were fitted to Gaussian distributions using "fit" function and "gauss" method (Curve Fitting Toolbox). Then, to extend the model to other daylengths, parameters were linearly interpolated or extrapolated using "interp1". Getting a Gaussian pulse for each gene dependent on daylength (equation 1).

$$input\ (t) = \alpha \ * e^{-\frac{(t-\beta)^2}{\gamma^2}} \qquad t \in [0, 24) \qquad \begin{cases} \beta = \beta\text{-}24; \ if\ t < \beta\text{-}12 \\ \beta = \beta; \ if\ \beta\text{-}12 \le t \le \beta + 12 \\ \beta = \beta + 24; \ if\ t > \beta + 12 \end{cases} \quad (1)$$

Representative Gaussian function per input gene repeated each day to generate Gaussian pulses, where "*α*", "*β*" and "*γ*" are positive real constants that define the peak: "*α*" amplitude (maximal expression), "*β*" center (phase - maximal expression time) and "*γ*" standard deviation (control width of expression). "*t*" denotes time. "*β*" value is modified by considering the closest center for each time point (*β*-24: day before, *β*: current day, *β*+24: next day) to simulate the pulses.

To simulate the 4-hour night extension following LD condition, *LHY2* input pattern was replaced by experimentally observed (Supplementary Figure 9; Supplementary Data 1).

FT2 pattern simulations

FT2 expression was described and predicted according to the ordinary differential equation 2, with its terms based on well-established gene regulatory network models[7]. Then, the models were simulated using "ode15s" function.

$$\frac{dFT2}{dt} = \frac{b + A}{1 + R1 + C + R2} - d * FT2$$

$$A = \frac{a * (ka_{GI} * GI)^{n_{GI}}}{(ka_{GI} * GI)^{n_{GI}}} \qquad R1 = r * (ka_{TOC1} * TOC1)^{n_{TOC1}}$$

$$C = (ka_{GI} * ka_{TOC1})^{(n_{GI} * n_{TOC1})} \qquad R2 = (K_{Repressor} * Repressor)^{n_{Repressor}} \quad (2)$$

"*b*" denotes basal transcription and "*d*" is the degradation rate. "A" represents the GI activation term, "R1" TOC1 repression, "C" GI-TOC1 competitive binding for occupancy of the same binding sites in *FT2* promoter, and "R2" repression by the second *"Repressor"*, which stands for LHY2

(Figure 3) or CDF2 (Supplementary Figure 7). Within these terms, "*a*" and *"r"* refer to the activation and repression rates, respectively. *"ka"* indices define the association affinities of the studied factors to *FT2* promoter. "*n*" are the Hill coefficients, a measurement of the degree of cooperativity of these associations. For "R2" term, since *"Repressor"* activity is linearly independent of the other elements, we can combine repression rate and association affinity into a single parameter, "$K_{Repressor}$", which states the repression strength. This relation is expressed as "$K_x = \sqrt[n_x]{a/r_x} * ka_x$". For the reader's reference, from the optimal parameter values listed in Supplementary Table 2, we can calculate this strength as K_{GI}= 2.6 and K_{TOC1}= 5.4; whereas K_{LHY2}= 4.2 and K_{CDF2}= 3.1.

Parameter value optimization

Hill coefficient parameters "*n*" were set to 3 based on evidence indicating that *Arabidopsis* orthologues form homo/heterocomplexes for DNA interactions, albeit specific complexes of action require further study[3,8-11]. The rest of the parameters were optimized using the simulated annealing algorithm ("simulannealbnd" function; Global Optimization Toolbox) to find a global minimum that fits best to the experimental observations. For that, qPCR patterns were smoothed to remove the single noisy data points using "fit" function and "smoothingspline" method (Curve Fitting Toolbox). Finally, a cost function was defined to minimize the Euclidean distance between the output of the model and the observed qPCR data (equation 3).

$$Cost = (Amplitude_{obs} - Amplitude_{sim})^2 + (Phase_{obs} - Phase_{sim})^2 + 0.05 * (Area_{obs} - Area_{sim})^2 + \sum_j (Point_{obs} - Point_{sim})^2 + 150 * \sum_k (Fold\ change_{obs} - Fold\ change_{sim})^2 \quad (3)$$

"*Amplitude*" refers to the maximal expression and "*Phase*" to the time of that maximum. "*Area*" represents the total amount of transcript accumulated during a day, measured using "trapz" function. "*Point*" denotes the transcript level at a specific time. "*Fold change*" is calculated by dividing the expression at ZT16 between indicated daylengths. "*obs*" means observed in the qPCR data fitted, and "*sim*" simulated. *j*=0,4,8,12,16,20 in ZT (hours from dawn). *k*=15h:13h/16h:12h, 14h:12h/15h:13h, 13h:11h/14h:12h (light hours:dark hours).

Gain and loss-of-function modeling

Altered genetic expressions were simulated by introducing the following modifications. To model knockout lines, expression of the corresponding gene was set to 1. In addition, for *lhy2* mutant, changes in GI and TOC1 patterns experimentally observed were considered after Gaussian fit, as described in Input genes section. For overexpressing lines driven by a constitutive promoter, an extra constant amount was consistently added all day long. TOC1 overexpression (TOC1ox) was simulated by adding either 50 or 0.6 units of transcript per minute, reproducing TOC1 ectopic expression level at ZT16 or aligning with *FT2* fold-change, respectively, as shown in Figure 2e. For GIox and LHY2ox 400 units of transcript per minute were added.

qPCR data used for model parameterization is attached as Supplementary Data 1, and a table including the parameter values is provided above as Supplementary Table 2.

SUPPLEMENTARY REFERENCES

[1] D. D. Seaton *et al.*, "Linked circadian outputs control elongation growth and flowering in response to photoperiod and temperature," *Mol Syst Biol*, vol. 11, no. 1, Jan. 2015, doi: 10.15252/msb.20145766.

[2] T. C. Mockler *et al.*, "The Diurnal Project: Diurnal and Circadian Expression Profiling, Model-based Pattern Matching, and Promoter Analysis," *Cold Spring Harb Symp Quant Biol*, vol. 72, no. 1, pp. 353–363, Jan. 2007, doi: 10.1101/sqb.2007.72.006.

[3] J. M. Gendron, J. L. Pruneda-Paz, C. J. Doherty, A. M. Gross, S. E. Kang, and S. A. Kay, "*Arabidopsis* circadian clock protein, TOC1, is a DNA-binding transcription factor," *Proceedings of the National Academy of Sciences*, vol. 109, no. 8, pp. 3167–3172, Feb. 2012, doi: 10.1073/pnas.1200355109.

[4] D. Dolfini, F. Zambelli, G. Pavesi, and R. Mantovani, "A perspective of promoter architecture from the CCAAT box," *Cell Cycle*, vol. 8, no. 24, pp. 4127–4137, Dec. 2009, doi: 10.4161/cc.8.24.10240.

[5] N. Gnesutta *et al.*, "CONSTANS Imparts DNA Sequence Specificity to the Histone Fold NF-YB/NF-YC Dimer," *Plant Cell*, vol. 29, no. 6, pp. 1516–1532, Jun. 2017, doi: 10.1105/tpc.16.00864.

[6] J. Wang *et al.*, "A major locus controls local adaptation and adaptive life history variation in a perennial plant," *Genome Biol*, vol. 19, no. 1, p. 72, Dec. 2018, doi: 10.1186/s13059-018-1444-y.

[8] R. Le Hir and C. Bellini, "The Plant-Specific Dof Transcription Factors Family: New Players Involved in Vascular System Development and Functioning in Arabidopsis," *Front Plant Sci*, vol. 4, 2013, doi: 10.3389/fpls.2013.00164.

[7] G. Karlebach and R. Shamir, "Modelling and analysis of gene regulatory networks", Nature reviews Molecular cell biology, vol. 9, no. 10, pp. 770-780, Oct. 2008, doi: 10.1038/nrm2503.

[9] S. X. Lu, S. M. Knowles, C. Andronis, M. S. Ong, and E. M. Tobin, "CIRCADIAN CLOCK ASSOCIATED1 and LATE ELONGATED HYPOCOTYL Function Synergistically in the Circadian Clock of Arabidopsis" *Plant Physiol*, vol. 150, no. 2, pp. 834–843, Jun. 2009, doi: 10.1104/pp.108.133272.

[10] J. Yan *et al.*, "TOC1 clock protein phosphorylation controls complex formation with NF-YB/C to repress hypocotyl growth," *EMBO J*, vol. 40, no. 24, Dec. 2021, doi: 10.15252/embj.2021108684.

[11] M. Sawa and S. A. Kay, "GIGANTEA directly activates *Flowering Locus T* in *Arabidopsis thaliana*," *Proceedings of the National Academy of Sciences*, vol. 108, no. 28, pp. 11698–11703, Jul. 2011, doi: 10.1073/pnas.1106771108.
