# Supplementary material for: Core clock genes adjust growth cessation time to day-night switches in poplar
Source: Nat Commun. 2024 Feb 27;15:1784. doi: 10.1038/s41467-024-46081-6 (PMC10899572; doi:10.1038/s41467-024-46081-6)
Supplement: Supplementary file 3 — Description of Additional Supplementary Files [file 41467_2024_46081_MOESM3_ESM.pdf]

Description of Additional Supplementary Files:

Supplementary Data 1: qPCR data generated for indicated daylengths and mutant scenarios.

Supplementary Data 2: MATLAB code for FT2 transcription model.

Supplementary Movie 1: Circadian phase shift dynamics downregulate FT2 expression transitioning to SD.
