# Supplementary material for: Core clock genes adjust growth cessation time to day-night switches in poplar
Source: Nat Commun. 2024 Feb 27;15:1784. doi: 10.1038/s41467-024-46081-6 (PMC10899572; doi:10.1038/s41467-024-46081-6)
Supplement: Supplementary file 5 — Supplementary Dataset 2 [file 41467_2024_46081_MOESM5_ESM.zip › Supplementary_File_2/README.rtf]

##########RUNNING SIMULATIONS#############################

TOC1-GI-LHY2 model can be simulated using MATLAB software (built on MATLAB_R2022a). To install MATLAB refer to https://es.mathworks.com/products/matlab.html (installation time ~15min).

To simulate it, run “run.m” file (running time few seconds). “run.m” takes as input:

-light: a number between 0 and 24 that defines the daylength in hours. It modulates the Gaussian pulses of the input genes (TOC1, GI, and LHY2) by linear interpolation between 12 and 16, or extrapolation outside of that range. 

-genotype: next genotypes can be simulated:

'WT': Wild type
'gi': GI knockout mutant
'GIox': GI constitutive overexpressor
'toc1': TOC1 knockout mutant
'TOC1ox': TOC1 constitutive overexpressor
'lhy2': LHY2 knockout mutant
'LHY2ox': LHY2 constitutive overexpressor

'Next': represents 4-hour night extension in LD and WT regime

(Any not valid genotype is taken as 'WT')

-gene: gene you want to plot as output. Only 'FT2' pattern is predicted. For 'GI', 'TOC1', and 'LHY2' the Gaussian pulse used as input is plotted.

(Any not valid gene is taken as 'FT2')  


*circaplot.m function was modified from Seaton et al., 2015

Seaton, Daniel D., et al. "Linked circadian outputs control elongation growth and flowering in response to photoperiod and temperature." Molecular systems biology 11.1 (2015): 776.
