# Supplementary figures and images for: Core clock genes adjust growth cessation time to day-night switches in poplar
Source: Nat Commun. 2024 Feb 27;15:1784. doi: 10.1038/s41467-024-46081-6 (PMC10899572; doi:10.1038/s41467-024-46081-6)

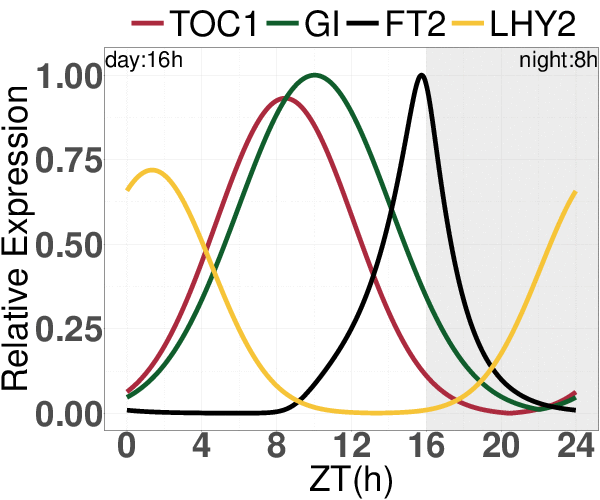

Supplement: Supplementary file 6 — Supplementary Movie 1 [file 41467_2024_46081_MOESM6_ESM.gif]
