# Supplementary material for: Core clock genes adjust growth cessation time to day-night switches in poplar
Source: Nat Commun. 2024 Feb 27;15:1784. doi: 10.1038/s41467-024-46081-6 (PMC10899572; doi:10.1038/s41467-024-46081-6)
Supplement: Supplementary file 7 — Reporting Summary [file 41467_2024_46081_MOESM7_ESM.pdf]

nature portfolio

Corresponding author(s): Krzysztof Wabnik
Mariano Perales

Last updated by author(s): Feb 1, 2024

Reporting Summary

Nature Portfolio wishes to improve the reproducibility of the work that we publish. This form provides structure for consistency and transparency in reporting. For further information on Nature Portfolio policies, see our Editorial Policies and the Editorial Policy Checklist.

Statistics

For all statistical analyses, confirm that the following items are present in the figure legend, table legend, main text, or Methods section.

n/a	Confirmed	
☐	☒	The exact sample size (*n*) for each experimental group/condition, given as a discrete number and unit of measurement
☐	☒	A statement on whether measurements were taken from distinct samples or whether the same sample was measured repeatedly
☐	☒	The statistical test(s) used AND whether they are one- or two-sided<br>*Only common tests should be described solely by name; describe more complex techniques in the Methods section.*
☒	☐	A description of all covariates tested
☐	☒	A description of any assumptions or corrections, such as tests of normality and adjustment for multiple comparisons
☐	☒	A full description of the statistical parameters including central tendency (e.g. means) or other basic estimates (e.g. regression coefficient) AND variation (e.g. standard deviation) or associated estimates of uncertainty (e.g. confidence intervals)
☐	☒	For null hypothesis testing, the test statistic (e.g. *F*, *t*, *r*) with confidence intervals, effect sizes, degrees of freedom and *P* value noted<br>*Give P values as exact values whenever suitable.*
☒	☐	For Bayesian analysis, information on the choice of priors and Markov chain Monte Carlo settings
☒	☐	For hierarchical and complex designs, identification of the appropriate level for tests and full reporting of outcomes
☒	☐	Estimates of effect sizes (e.g. Cohen's *d*, Pearson's *r*), indicating how they were calculated

Our web collection on statistics for biologists contains articles on many of the points above.

Software and code

Policy information about availability of computer code

Data collection	-Software integrated into Roche LightCycler 480 II instrument (Roche Diagnostics, Barcelona, España). Relative quantification option was used to obtain Ct values in qPCR experiments.
Data analysis	-ClustalW multiple aligment tool in BioEdit Sequence Aligment Editor 7.0 was used for DNA and protein aligments.<br>-R version 4.03 was used for data graphics and statistical analysis.<br>-MATLAB_R2022a was applied for qPCR data processing and modeling.

For manuscripts utilizing custom algorithms or software that are central to the research but not yet described in published literature, software must be made available to editors and reviewers. We strongly encourage code deposition in a community repository (e.g. GitHub). See the Nature Portfolio guidelines for submitting code & software for further information.

Data

Policy information about availability of data

All manuscripts must include a data availability statement. This statement should provide the following information, where applicable:

- Accession codes, unique identifiers, or web links for publicly available datasets
- A description of any restrictions on data availability
- For clinical datasets or third party data, please ensure that the statement adheres to our policy

All data supporting the findings of this study are available within the article and its Supplementary Information. Raw values generated in this study are provided in

the Source Data file. Poplar accession numbers are provided in Methods section. Raw values for Arabidopsis thaliana daily patterns of transcription (Supplementary Figure 3) were obtained from the microarray datasets "long-day" and "short-day" available in DIURNAL database from Mockler Lab (http://diurnal.mocklerlab.org/diurnal_data_finders/new).

Research involving human participants, their data, or biological material

Policy information about studies with human participants or human data. See also policy information about sex, gender (identity/presentation), and sexual orientation and race, ethnicity and racism.

Reporting on sex and gender	This information has not been collected.
Reporting on race, ethnicity, or other socially relevant groupings	n/a
Population characteristics	n/a
Recruitment	n/a
Ethics oversight	n/a

Note that full information on the approval of the study protocol must also be provided in the manuscript.

Field-specific reporting

Please select the one below that is the best fit for your research. If you are not sure, read the appropriate sections before making your selection.

☒ Life sciences ☐ Behavioural & social sciences ☐ Ecological, evolutionary & environmental sciences

For a reference copy of the document with all sections, see nature.com/documents/nr-reporting-summary-flat.pdf

Life sciences study design

All studies must disclose on these points even when the disclosure is negative.

Sample size	Sample sizes were chosen based on last accepted guidelines for working with poplar plants (doi.org/10.1038/s41467-021-21449-0; doi.org/10.1038/s41467-018-06696-y; doi.org/10.1016/j.cub.2019.06.003; doi.org/10.1111/nph.15087), aiming to strike a balance between result credibility and the space- and time-consuming nature of sample processing. For experiments with a limited number of collection points, we established an n=4/6, while for time-course experiments with several data points, n=2. Low deviations in our results indicated to us that this sample sizes are reliable and, then, sufficient.
Data exclusions	No data were excluded from the analyses
Replication	Each experimental assay was repeated at least twice to ensure that plant growth conditions did not have an impact on reproducibility. Furthermore, for qPCR analyses, each biological replicate results from the arithmetic mean of three technical replicates with virtually no deviation to minimize procedural and human errors.
Randomization	Poplar genotypes were randomly arranged in growth chambers before the experiments were conducted, and the timing of sample collection was also randomized among the lines under study.
Blinding	Plants were cultivated under highly controlled and homogeneous conditions (except for the photoperiod cue, as it is the matter of this study) in growth chambers. And, as stated above, subsequent analyses were conducted using biological and technical replicates, so blinding was not necessary.

Reporting for specific materials, systems and methods

We require information from authors about some types of materials, experimental systems and methods used in many studies. Here, indicate whether each material, system or method listed is relevant to your study. If you are not sure if a list item applies to your research, read the appropriate section before selecting a response.

Materials & experimental systems

n/a	Involved in the study	
☒	☐	Antibodies
☒	☐	Eukaryotic cell lines
☒	☐	Palaeontology and archaeology
☒	☐	Animals and other organisms
☒	☐	Clinical data
☒	☐	Dual use research of concern
☐	☒	Plants

Methods

n/a	Involved in the study	
☒	☐	ChIP-seq
☒	☐	Flow cytometry
☒	☐	MRI-based neuroimaging

Dual use research of concern

Policy information about dual use research of concern

Hazards

Could the accidental, deliberate or reckless misuse of agents or technologies generated in the work, or the application of information presented in the manuscript, pose a threat to:

No	Yes	
☒	☐	Public health
☒	☐	National security
☒	☐	Crops and/or livestock
☒	☐	Ecosystems
☒	☐	Any other significant area

Experiments of concern

Does the work involve any of these experiments of concern:

No	Yes	
☒	☐	Demonstrate how to render a vaccine ineffective
☒	☐	Confer resistance to therapeutically useful antibiotics or antiviral agents
☒	☐	Enhance the virulence of a pathogen or render a nonpathogen virulent
☒	☐	Increase transmissibility of a pathogen
☒	☐	Alter the host range of a pathogen
☒	☐	Enable evasion of diagnostic/detection modalities
☒	☐	Enable the weaponization of a biological agent or toxin
☒	☐	Any other potentially harmful combination of experiments and agents

Plants

Seed stocks	All poplar lines experimentally tested were generated in the lab, either previously or for this study as indicated in the article.
Novel plant genotypes	New poplar loss-of-function lines were produced using CRISPR-Cas9 technology. sg RNAs targeting the gene of interest were selected from a pre-designed SNP-free dataset available on AspenDB (https://www.aspendb.org/) as shown in Supplementary Fig. 4. Agrobacterium tumefaciens strain GV3101/pMP90, carrying the p201N-Cas9 vector with the appropriate sgRNA, was employed for transformation. More than 10 independent lines were screened for positive mutation. A representative line for each gene was used for subsequent experiments and is presented in this article.
Authentication	CRISPR-Cas9 loss-of-function lines were assessed by sequencing the target region of the gene of interest. Positive mutants were confirmed after predicting non-functional protein truncation for both haplotypes in our poplar system (P. tremula x P. alba), as indicated in Methods.
